# Evolutionary Dynamics in the Southwest Indian Ocean Marine Biodiversity Hotspot: A Perspective from the Rocky Shore Gastropod Genus *Nerita*

**DOI:** 10.1371/journal.pone.0095040

**Published:** 2014-04-15

**Authors:** Bautisse Postaire, J. Henrich Bruggemann, Hélène Magalon, Baptiste Faure

**Affiliations:** 1 Laboratoire d’ECOlogie MARine, Université de la Réunion, FRE3560 INEE-CNRS, Saint Denis, La Réunion, France; 2 Labex CORAIL, Perpignan, France; 3 Biotope, Service Recherche et Développement, Mèze, France; University of Canterbury, New Zealand

## Abstract

The Southwest Indian Ocean (SWIO) is a striking marine biodiversity hotspot. Coral reefs in this region host a high proportion of endemics compared to total species richness and they are particularly threatened by human activities. The island archipelagos with their diverse marine habitats constitute a natural laboratory for studying diversification processes. Rocky shores in the SWIO region have remained understudied. This habitat presents a high diversity of molluscs, in particular gastropods. To explore the role of climatic and geological factors in lineage diversification within the genus *Nerita*, we constructed a new phylogeny with an associated chronogram from two mitochondrial genes [cytochrome oxidase sub-unit 1 and 16S rRNA], combining previously published and new data from eight species sampled throughout the region. All species from the SWIO originated less than 20 Ma ago, their closest extant relatives living in the Indo-Australian Archipelago (IAA). Furthermore, the SWIO clades within species with Indo-Pacific distribution ranges are quite recent, less than 5 Ma. These results suggest that the regional diversification of *Nerita* is closely linked to tectonic events in the SWIO region. The Reunion mantle plume head reached Earth’s surface 67 Ma and has been stable and active since then, generating island archipelagos, some of which are partly below sea level today. Since the Miocene, sea-level fluctuations have intermittently created new rocky shore habitats. These represent ephemeral stepping-stones, which have likely facilitated repeated colonization by intertidal gastropods, like *Nerita* populations from the IAA, leading to allopatric speciation. This highlights the importance of taking into account past climatic and geological factors when studying diversification of highly dispersive tropical marine species. It also underlines the unique history of the marine biodiversity of the SWIO region.

## Introduction

The heterogeneity of species distribution is primarily due to latitudinal, longitudinal and altitudinal gradients in environmental conditions [1]. However, such gradients do not explain the presence of highly species-rich regions, or “biodiversity hotspots”. Marine and terrestrial biodiversity hotspots have been defined as restricted regions with high species richness, high endemism and facing habitat loss due to human activities [2], [3]. Over the past two decades, 34 terrestrial et 10 marine biodiversity hotspots have been identified [2], [4]. The Indo-Australian archipelago (IAA), as defined by [5], represents the most important marine biodiversity hotspot, with species richness declining gradually westward and eastward [6], [7]. Two main theories have been proposed to explain this distribution of marine biodiversity: the centre-of-origin and the centre-of-overlap hypothesis [8]. The first proposes that this region generates novel species arising in sympatry or allopatry between islands that subsequently migrate outwards to peripheral regions. The second theory proposes that the IAA, located at the limit of several biogeographical regions, accumulates species formed by allopatric speciation outside the IAA. It should be noted that these hypotheses are not mutually exclusive and patterns may vary among taxa (reviewed in [9]).

Shallow marine habitats are strongly affected by tectonics, eustatic sea-level changes, physical disturbances and run-off from land, impacts that continually modify habitat availability over various time scales [10], [11]. Furthermore, climatic variation over geologic time changes environmental conditions and ocean currents at global and regional scales [10]. These abiotic changes alter gene flow and species distribution and may eventually lead to the formation of new species due to vicariance and/or new selective pressures [12]. Several studies argued that part of the origin of the IAA marine hotspot could be explained by abiotic factors, such as sea level fluctuation and oceanic circulation, tectonic activity and temperature changes [13]–[18]. Recent studies further pointed out the role of habitat, with coral reefs enhancing species diversification [17], [19].

The Southwest Indian Ocean (SWIO) region, corresponding to the Western Indian marine ecoregion [20] comprises a main landmass, Madagascar, and several island archipelagos such as Comoros, Mascarenes and Seychelles, each with different origins and ages. While Madagascar and the Seychelles are fragments of eastern Gondwana, the Mascarene archipelago was formed during the past 10 Ma by the volcanic activity of a mantle plume, which has been active since the late Cretaceous [21]. This region hosts a high proportion of endemics and is highly threatened by human activities, hence its classification as a marine biodiversity hotspot [2], [22]. It further is one of the regions where terrestrial and marine biodiversity hotspots coincide. The study of the SWIO terrestrial biota revealed a high relatedness between SWIO and Asian species, leading to the hypothesis of the presence of discontinuous land bridges connecting islands and continents to explain present species distributions [23]–[25]. The extent of such ephemeral bridges varied with sea level and is thought to have facilitated the colonisation of SWIO by terrestrial species following a stepping-stone model [24], [26], [27]. The study of SWIO marine taxa, focusing on reef-associated species, also highlighted the relatedness of SWIO and IAA faunas, but also revealed the presence of numerous cryptic species [8], [17], [28], [29]. In contrast, a recent study argued that the northern Mozambique Channel hosts scleractinian corals that are relicts of the West Tethys biodiversity [30]. While these studies revealed the complex and contrasted evolutionary histories of SWIO marine taxa, they concerned mainly reef-associated biota, leaving aside other marine ecosystems.

Rocky shores represent only 3000 km^2^ area in the Indian Ocean (excluding the Western Australian coast) [31]. In spite of the limited extent, this habitat is extremely interesting for studying species evolution. Rocky shores are characterised by strong gradients in environmental conditions at small scales, varying within meters and hours, and so exert strong selection pressures enhancing species diversification over time [32]. Several biogeographical studies revealed the high variability of genetic structures and speciation patterns of rocky shore species, with some taxa showing only weak imprint of past variations in environmental conditions while others displaying distribution patterns that are highly correlated to the geological history [33]–[35]. Differential dispersal capacities provides an intuitive explanation for these discrepancies among species: population structure of species with long-lived planktonic larva show little imprint of environmental changes due to high connectivity, as speciation occurs at ocean basin scales and exclusively by allopatric processes [36]. However, attributing the observed biogeographic patterns and population genetic structures to the specificities of larval life alone is too simplistic, as the ecological requirements of the adult stage also determine the distribution of marine species [37], [38]. Knowing the geographic range, the ecological requirements and life history traits, in addition to geological history, is challenging but often necessary to accurately resolve the evolutionary history of a species.

**Figure 1:**
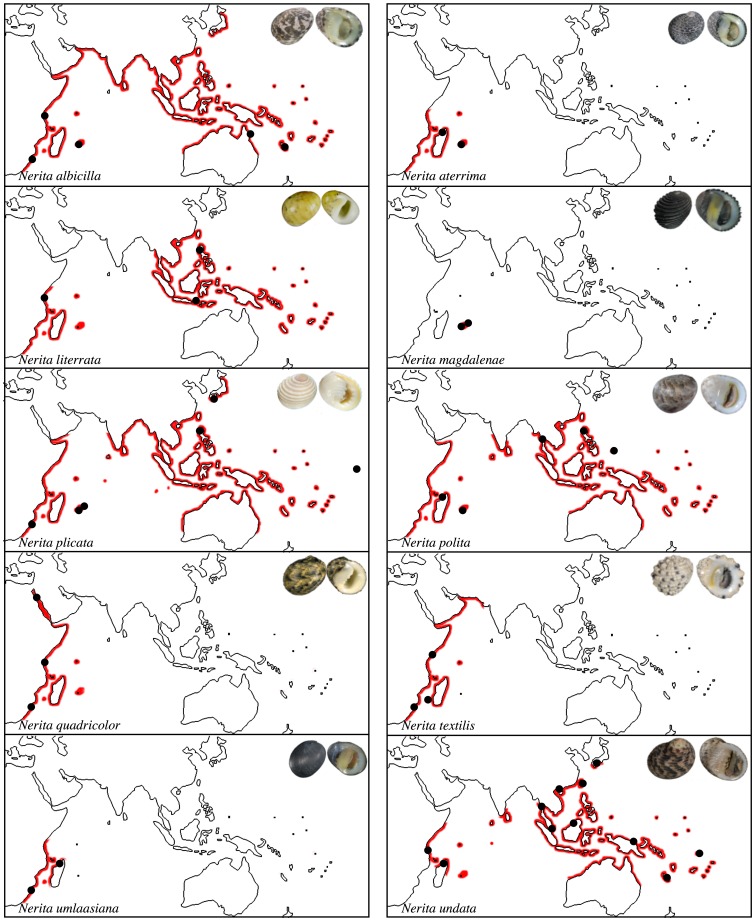
Geographic distribution of *Nerita* species living in the South-Western Indian Ocean, adapted from [16]. Grey dots indicate sampling sites from [16]; grey squares indicate sampling sites of new haplotypes.

The Neritidae (Rafinesque) (Neritimorpha [39]) family is composed of tropical gastropods inhabiting a wide variety of habitats: open seawater, rocky shores, brackish and fresh waters, mangroves, mud and sand. However, the genus *Nerita* (Linnaeus), with more than 60 known extant species is almost restricted to tropical rocky shores and has already been subject to phylogeographic studies [16], [40], [41]. Niche changes have played a relatively minor role in the diversification of the genus [16]. Species diversity in this genus peaks in the IAA region, but many species present large biogeographical ranges, from the SWIO to Hawaii [16]. Members of the genus have planktotrophic larvae that live for weeks up to months before settling [42], [43]. Those long-lived larvae are expected to limit the number of regional endemics due to high connectivity between populations, which is generally observed in *Nerita*. Contrasting with this general pattern, the SWIO rocky shores present a high proportion of endemics. Among the ten *Nerita* species present, one is endemic to the Mascarene archipelago, *N. magdalenae* (Gmelin), two are endemic to the SWIO, *N. aterrima* (Gmelin) and *N. umlaasiana* (Krauss), while two are restrained to the western coasts of the Indian Ocean, *N. textilis* (Gmelin) and *N. quadricolor* (Gmelin) ([16], Figure 1). The phylogeny of the genus is almost resolved and several clades restricted to the SWIO have been identified within species presenting an Indo-Pacific distribution. Further study of the diversification of the genus have set its origin at the end of the Paleocene and showed a constant diversification rate since then [38]. According to this study, species living in the SWIO originated during two distinct periods, at the beginning (±22 Ma; *N. magdalenae, N. textilis* and *N. aterrima*) and the end of the Miocene (±9 Ma; *N. quadricolor* and *N. umlaasiana*).

Time-calibrated phylogenies contain information on the temporal diversification of clades, permitting the exploration of macroevolutionary processes such as variation of speciation and extinction rates over time [44]. When biogeographical information of extant lineages is also considered, a chronogram allows understanding how past geological and climatic events influenced present-day species richness and distribution patterns [45]. According to [40], the IAA is a centre-of-overlap, conclusion supported by the observation that no recent lineage of *Nerita* species has originated within this region. However, this study did not fully explore the diversification patterns of SWIO *Nerita* species and clades.

The present study focuses on the origins and diversification of SWIO *Nerita* species and complements previous phylogenies. It participates to the global effort in understanding how the unique biodiversity of the SWIO region appeared and was maintained through geological times. Many questions remain unanswered: Are local species formed via regional or more global speciation processes? Do they present trans-ocean origins? Are they ancient or relatively recent species? To answer such questions, we used up-to-date methods of chronogram reconstruction and macroevolution studies to (1) reveal variations in the diversification history of *Nerita* species, and (2) study the potential influence of extrinsic geological and climatic factors on the diversification of the genus.

## Materials and Methods

### Sampling

We thank the Affaires Maritimes, the Réserve Naturelle Marine de La Réunion and the Territoires Australes et Antarctiques Françaises for sampling permits and the latter also for logistic support during fieldwork at the Eparse Islands. We collected shells and tissues of eight *Nerita* morpho-species from different locations within the SWIO (32 individuals in total, Table 1, Figure 1). Species were identified *a posteriori* using online databases and taxonomic works [46], [47]. To detect possible cryptic species, we collected and sequenced at least three individuals per morpho-species. Samples from Madagascar and Europa Island were collected between 2008 and 2010 and samples from Reunion and Mauritius were collected in 2011. Each individual (shell) was photographed alive. Samples previous to 2010 were stored in 90% ethanol at room temperature and samples of later campaigns were preserved after dissection in 70% ethanol and stored at −20°C.

**Table 1:** Number of newly sampled specimens per location.

	Reunion Island	Mauritius	Europa Island	Madagascar
*Nerita albicilla* (Linnaeus 1758)	4			
*Nerita aterrima* (Gmelin 1791)	6			
*Nerita magdalenae* (Gmelin 1791)	3	2		
*Nerita plicata* (Linnaeus 1758)	6	2		
*Nerita polita* (Linnaeus 1758)	3			
*Nerita quadricolor* (Gmelin 1791)			3	
*Nerita textilis* (Gmelin 1791)			2	
*Nerita umlaasiana* (Krauss 1848)				1

Each specimen was sequenced for both markers (CO1 and 16S).

### Sequencing

DNA of each individual was extracted from ∼10 mg of foot tissue with DNeasy Blood & Tissue Kit (Qiagen) following the manufacturer’s protocol. Extraction quality was assessed visually on a 0.8% agarose gel stained with GelRed Nucleic Acid Stain, 10000X in DMSO (Gentaur). We amplified and sequenced fragments of two mitochondrial genes, cytochrome oxidase sub-unit 1 (CO1) and 16 s rRNA (16S). Each PCR reaction was conducted in 25 µl: 8,5 µl of ultra-pure water, 12.5 µl (0.625U) of AmpliTaq mix (Applied Biosystems), 1 µl of each primer (10 µM) and 2 µl of template DNA (final concentration: 1.6 ng/µl). Each gene was amplified with the following sets of primers: CO1 with HCO2198 (5′-TAA ACT TCA GGG TGA CCA AAA AAT CA-3′) and LCO1490 (5′-GGT CAA CAA ATC ATA AAG ATA TTG G-3′) [48] and 16S with 16Sar (5′-CGC CTG TTT ATC AAA AAC AT-3′) and 16Sbr (5′-CCG GTC TGA ACT CAG ATC ACG T-3′) [49]. The PCR profile for both markers consisted of an initial denaturation step (5 min at 95°C), 5 cycles of denaturation (30 s at 94°C), annealing (30 s at 46°C) and elongation (1 min at 72°C), 30 cycles of denaturation (30S at 94°C), annealing (30 s at 51°C) and elongation (1 min at 72°C), and a final extension step (10 min at 72°C). PCR products were visualized on a 1% agarose gel stained with GelRed Nucleic Acid Stain, 10000X in DMSO (Gentaur). PCR products were sequenced in both directions at Genoscreen (www.genoscreen.fr) by the Sanger method (3730XL, Applied Biosystems).

### Tree Construction

The 64 new sequences were checked and edited using Geneious 6.0 (created by Biomatters, available on from http://www.geneious.com/) and deposited in Genbank (Table S1). To complete our data set, sequences for CO1 and 16S genes were downloaded from Genbank (Table S1). In fine, 370 *Nerita* sequences were used (185 for each marker). The complete data set was aligned using the ClustalW algorithm (default options) and confirmed by eye. Using Modeltest v3.7 [50], [51] and AIC, GTR+I+Γ was the best evolution model. Lack of significant divergence in the phylogenies of the two markers permitted conducting the analysis on the combined data set [40]. Three methods of phylogenetic tree construction were used: (1) Neighbor-joining (NJ, 1000 bootstrap permutations) was calculated using Geneious 6.0 (2) Maximum Likelihood (ML) was implemented using the PHYML Geneious plug-in [52] and (3) Bayesian analyses were performed using MrBayes v3.2 (MB, 30.10^6^ generations, 8 chains, 3 runs, temperature to 0.2) [53]. Bayesian analyses were conducted on Titan, Reunion University’s cluster. Each tree was rooted by the outgroup *Bathynerita naticoidea* [54].

### Estimation of Nodes Ages

We used BEAST v1.7.5 [55] to reanalyse the combined dataset of CO1 and 16S sequences incorporating an uncorrelated relaxed, lognormal molecular clock for both markers. This method allows the construction of phylogeny and estimation of divergences times at the same time, calculating the 95% Highest Posterior Density (HPD) of each node. The chronogram was calibrated as in [40]. We used a GTR+Γ+I substitution model for each marker. We used the birth-death model for the tree reconstruction [56], which assumes constant speciation and extinction rates over time (exponential increase of species number over time) with a faster accumulation of lineages when considering recent past geological time (accumulation of lineages that are not extinct yet).

Analyses were undertaken with three independent chains of 1.10^8^ generations, sampling every 10^4^ generations. The convergence of all parameters was assessed using Tracer v1.5 [57]. Final tree was produced by constructing a consensus tree from the data set of accepted trees (burnin: 25%, total accepted: 7500) with the maximum clade credibility option and median node height using TreeAnnotator v1.7.5 [58]. Final tree editing was performed using FigTree v1.4 [59].

### Diversification Analysis

#### Tree imbalance assessment

We assessed the imbalance of the tree using β. The β parameter compares the observed nodal imbalance among clades to the equal-rates Markov model [60]. Under the theoretical model, each node has the same probability of splitting, and β should not be distinguishable from 0. Strong negative or positive values indicate variations in the splitting probability among lineages indicating whether lineages within a tree have diversified with different rates [60]. We performed this analysis for the whole genus using *apTreeshape* [61] package under R [62].

To explore the evolution of the genus through time, lineage-through-time (LTT) plots were constructed. Based on the chronogram, it represents the evolution of the number of lineages against the node ages and thus the change in diversification rate through time. One thousand random trees were calculated with the same parameters (root height, number of taxa, diversification and extinction rates) as our data to assess a null distribution of LTT curves. Under a constant birth-death model, a straight line is expected with slope equal to b–d (b: speciation, d: extinction) [56], [63].

#### Diversification rate shifts

Departures from a constant rate of diversification (null hypothesis) were analysed using the constant rate test. This test uses the gamma (γ) statistic to compare the positions of nodes of the studied phylogeny to those from a theoretical phylogeny generated under a constant rate of diversification [63] using the package *ape* [64]. Negative values of γ imply a reduction of the speed of accumulation of lineages and thus of diversification rate; positive values imply an acceleration of lineage accumulation. The gamma statistic assumes that all extant lineages have been sampled and that speciation and extinction occur with equal probability among lineages. To account for incomplete sampling, we used the MCCR test [63]. We simulated a thousand phylogenies with 100 taxa under the pure birth model. We compared the γ value of the original phylogeny to the distribution of values of the random phylogenies. To test the second assumption of the constant rate test, we used the relative cladogenesis (RC) test with a Bonferroni correction implemented the *geiger* package [65]. This test calculates the probability that a particular lineage existing at a time t will have n descendants under a constant rate birth-death model at the present time. The null hypothesis of this test is that all ancient branches have the same proportion of extant species [66]. It allows identifying branches producing more descendant than the others at the same time interval.

When using phylogenies with a non-null extinction rate [67], the power of the γ statistic for detecting changes in diversification rates is lower than likelihood methods (e.g., ΔAIC_RC_) (Table S2). The ΔAIC_RC_ test statistic included in the *laser* package [68] compares data fits to rate-constant diversification models (Yule and birth-death, AIC_RC_) and several rate-variable models (Yule-2-rate, Yule-3-rate, logistic density-dependent DDL and exponential density-dependent DDX, AIC_RV_) with likelihood ratio tests and AIC scores. Under DDL model, the speciation rate slows in relation to a logistic growth depending on: the initial speciation rate, the number of lineages at a specific point and a parameter similar to the carrying capacity. Under the DDX model, the speciation rate at a certain point of time is function of initial speciation rate, the number of lineage at this specific point and the magnitude of the rate change as the number of lineage increases (a rate change equals to 0 implies constant speciation through time) [69]. In the other two rate-variable models, the speciation rate under a pure birth model within a clade is assumed to change before one or several breakpoints [68]. For example, the Yule-2-rate assumes a first speciation rate, a final speciation rate, and inserts a breakpoint in time when rate shifts (optimized during model fitting). According to [67], rate-constant models can be rejected with confidence when the difference between AIC_RC_ and AIC_RV_ is close to 4 for small phylogenies (n_taxa_ = 30) and 5.5 for large phylogenies (n_taxa_ = 100). We assessed the critical values of the ΔAIC_RC_ test by simulating 1000 phylogenies of 74 taxa under the pure birth model (Table S3).

We used the *TreePar* package 2.5 [70] to highlight changes in diversification rates (speciation-extinction), i.e. the slope of the LTT, through time by adjusting the starting time of the analysis and taking into account incomplete sampling. The control of these two parameters allows controlling the possibility that recent evolution and incomplete sampling may hide diversification phases during the early stages of the phylogeny. The sampling parameter represents the proportion of extant lineages present in the phylogeny (this parameter set to 1 indicates that all lineages have been sampled). This method needs the specification of the boundaries of the period studied (here from 34 to 3 Ma, covering Oligocene, Miocene and Pliocene), the grid (here 1 Ma) and the number of shifts to include. On each point of the grid, a shift is inserted and rates are estimated. Once the best shift point is determined, it is fixed and the analysis restarts to insert another shift point. Likelihood ratio tests were used to compare the current model to a model with one more shift point.

#### Sliding window analysis

In order to explore potential bursts of diversification through time, we performed a sliding-window analysis [71]. Assuming that all non-sampled lineages were formed during the Pleistocene (e.g. 2.6 Ma to Present), we set a window of 5 Ma wide and a step of 1 Ma, and calculated a diversification rate for each step using the chronogram from BEAST from 56 to 2.6 Ma. In [72], the diversification rate is given as –ln (Nb/Nt)/Δt, where Nb is the number of lineages at the beginning of the period, Nt the number of lineages at the end of the period and Δt the length of the period in million years.

## Results

### Molecular Dating

**Figure 2:**
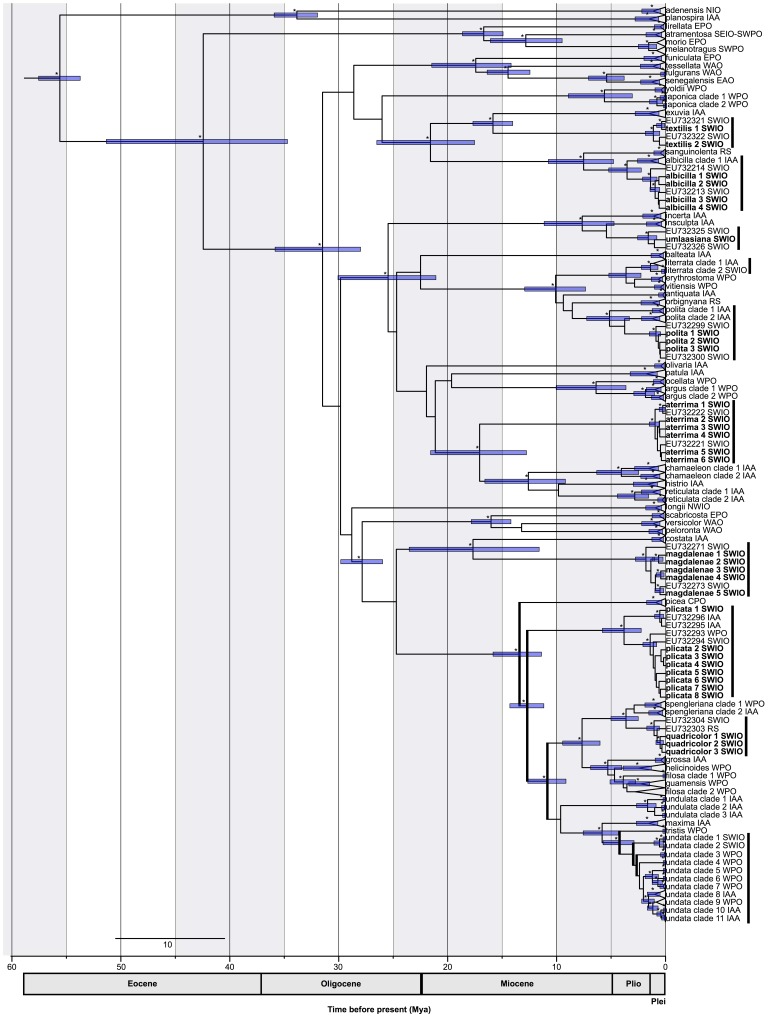
Maximum clade credibility tree. Branch lengths are proportional to time in million years (Ma) and are estimated with Bayesian relaxed lognormal molecular clocks for each marker on the concatenated dataset. Clades with significant increase of diversification rates are indicated by thick black lines (p<0.05). Asterisks on branches represent clades supported by the Bayesian analysis (posterior probability >0.95). Bars represent the 95% HPD interval around the dated nodes. NWIO: Northwest Indian Ocean; SEIO: Southeast Indian Ocean; SWIO: Southwest Indian Ocean; IAA: Indo-Australian archipelago; SWPO: Southwest Pacific Ocean; WPO: West Pacific Ocean; CPO: Central Pacific Ocean; EPO: East Pacific Ocean; RS: Red Sea; WAO: West Atlantic Ocean; EAO: East Atlantic Ocean. Each triangle tips represent two sequences from Genbank. Bold names represent new sequences. Thick black lines identify species present in the SWIO.

Our phylogenetic analysis was fully consistent with the previous published phylogeny of *Nerita* [40]. No new clades within *Nerita* species were discovered. The tree generated with an uncorrelated relaxed lognormal molecular clock for both markers indicates divergence times (Figure 2). The relaxed molecular clock estimated that the genus *Nerita* originated 55.7 Ma ago (95% HPD: 53.7 to 57.6 Ma). Even though some nodes are poorly supported, the majority of nodes ages were estimated with high confidence. Our chronogram, using two independent molecular clocks, dated all diversification events more recently than previous findings, though with the same topology and confidence of nodes. Estimated ages of *Nerita* species present in the SWIO region are all younger than 20 Ma: *N. aterrima* appeared 17.26 Ma (95% HPD: 12.76 to 22.71 Ma); *N. magdalenae* 17.9 Ma (95% HPD: 11.27 to 23.8 Ma); *N. quadricolor* 3.59 Ma (95% HPD: 2.4 to 4.99 Ma); *N. textilis* 15.86 Ma (95% HPD: 13.99 to 17.65 Ma); *N. umlaasiana* 5.39 Ma (no 95% HPD given by BEAST). For species with Indo-Pacific range, individuals collected in the SWIO show divergent and strongly supported monophyletic clades (except *N. polita*): *N. albicilla* SWIO diverged from the IAA clade 3.64 Ma (95% HPD: 2.18 to 5.63 Ma); *N. polita* 3.73 Ma (no 95% HPD given by BEAST); *N. litterata* 1.13 Ma (no 95% HPD given by BEAST); two *N. undata* clades 2.67 Ma (no 95% HPD given by BEAST).

### Evolution of Lineages and Tree Shape

The chronogram obtained with BEAST suggested imbalance in the diversification of *Nerita* as it is more imbalanced than expected under the Yule model (β = −1.07, 95% confidence interval: −1.43 to −0.56). The RC test highlighted an increase in diversification rate starting at 13.15 Ma (95% HPD: 11.39 to 15.83 Ma, p = 0.0076, α = 0.05) and running all along the phylogeny to the *N. undata* species complex.

Studies of diversification rate shifts through time selected the Yule-3-rate model as the best fit model for the whole *Nerita*, but did not reject the Yule model (γ = 0.63; critical value γ = 1.96, α = 0.05).

The *TreePar* analyses detected a reduction of diversification rates situated at 24 Ma, decreasing from 0.058 to 0.00013 (Table 2).

**Table 2:** Determination of the number of rate shifts for *Nerita*.

Rs	T0	Tu	Div	T1	Tu	Div	T2	Tu	Div	p value	Log-likelihood
0	0	0.49	0.068								−204.656
1	0	0.99	0.00013	24	0.57	0.058				0.0403	−202.554
2	0	0.98	0.0042	6	0.18	0.006	24	6.10^−7^	0.13	0.0709	−200.923

Log-likelihood values were tested against the model with one more shift. Rs : rate shifts, Tn : n rate shift, Tu : turnover, Div: diversification.

**Figure 3:**
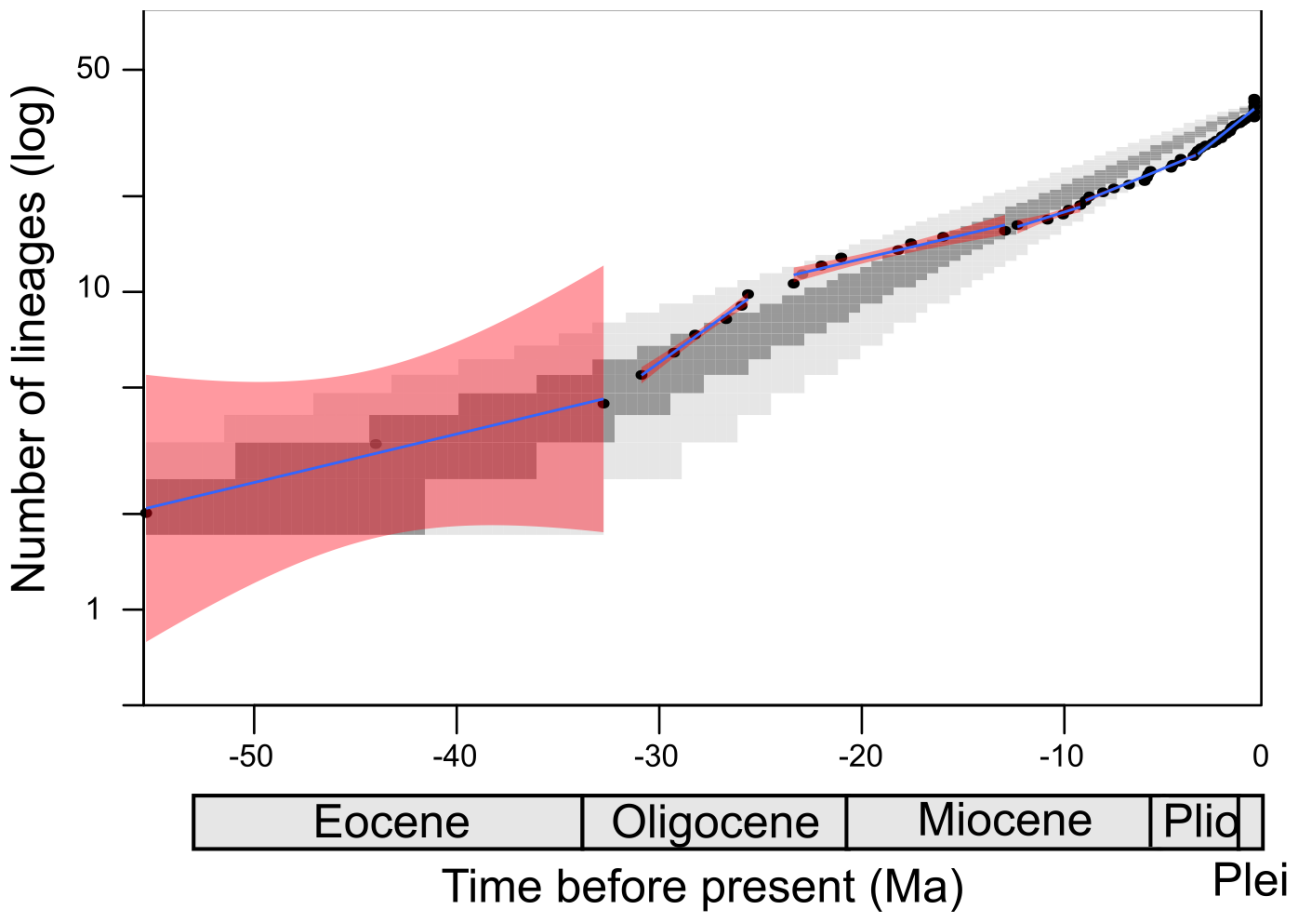
LTT plot of the maximum clade credibility tree. Grey zone represent the 50% (dark grey) and 95% (light grey) null distribution of LTT plots generated under the Yule model. Black dots represent observed values. Blue lines represent successive linear models fitted to the data and confidence intervals (red).

The LTT plot is consistent with the *TreePar* analyses, showing a faster accumulation of lineages during the Oligocene and a later slowdown (Figure 3, Table 3). However, the LTT plot remains within the 95% confidence interval of null distributions of LTT plots under the Yule model.

**Table 3:** Parameters of the linear models fitted to the LTT for each geological period.

Geological period (in Ma)	Slope (lineage per Ma)	Intercept
Eocene to early Oligocene (−50, −30)	**1.11**	0.020
Early Oligocene to early Miocene (−30, −20)	2.44	0.059
Early Miocene to mid-Miocene (−20, −15)	1.79	0.036
Mid-Miocene to late Miocene (−15, −10)	1.60	0.023
Late Miocene (−10, −5)	1.63	0.026
Pliocene to nowadays (−5, 0)	1.77	0.051

**Figure 4:**
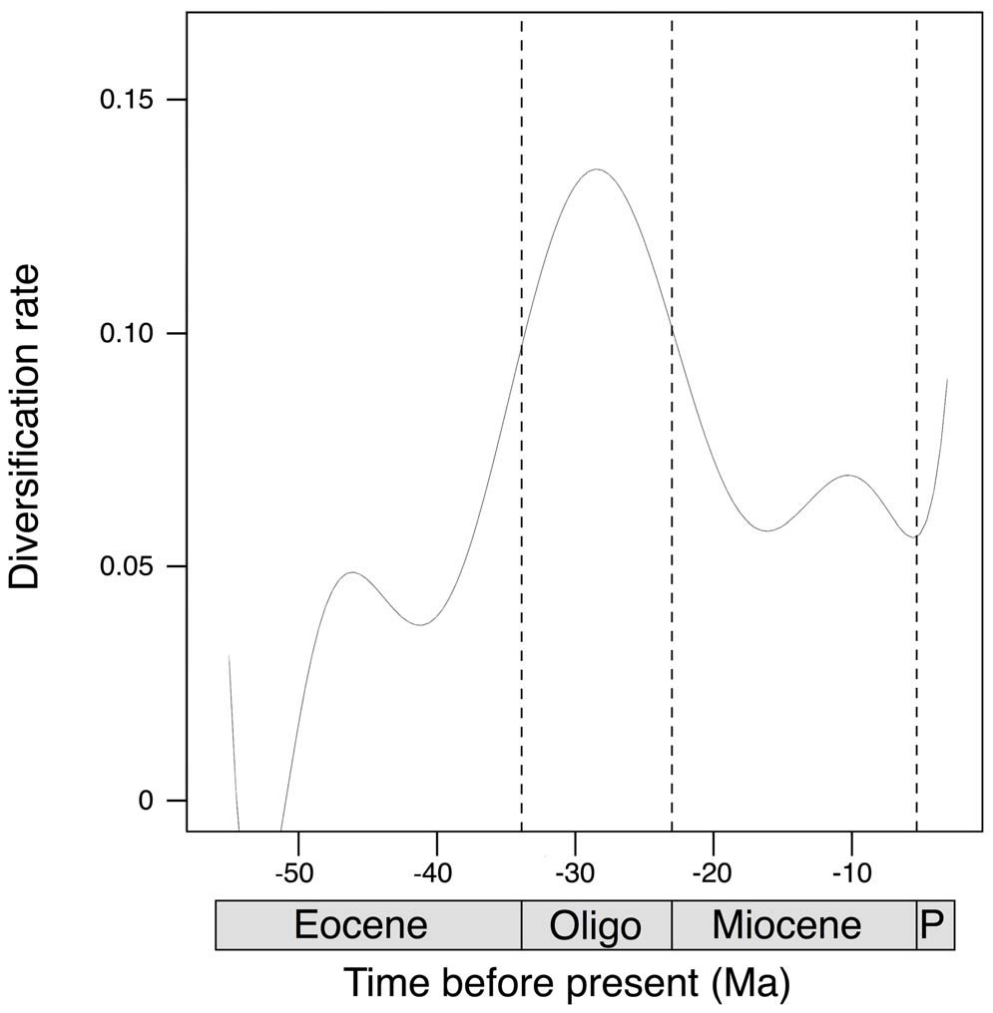
Sliding-window analysis of the net diversification rate of the genus *Nerita* through geological periods. Oligo: Oligocene; P: Pliocene.

The sliding-window analysis is also consistent with previous results, showing that the diversification rate was higher during the Oligocene. It decreased during the Miocene and remained relatively constant for 10 Ma (Figure 4) to finally increase again during more recent geological times.

## Discussion

### Biogeography: Global Patterns and Timing of Diversification

Our results provide another example of extensive cryptic diversity in species with Indo-Pacific distribution ranges. *Nerita* species from IAA present numerous robust divergent clades within this region. This pattern has been interpreted as a consequence of intense diversification within this region, producing species that further disperse and colonize peripheral islands in the Indian and Pacific oceans [73]. Long distance colonization events may lead to allopatric and peripatric speciation with the modification of gene flow through time due to changes in biotic and abiotic conditions. Allopatry seems to be the most frequent speciation mechanism of marine species [28], [33], [37], [74], but other processes like disruptive selection, habitat or resource choice, may occur at smaller geographic scales and lead to sympatric sister species, particularly in gastropods [75].

The first fossil record of *Nerita* is from the late Paleocene (56 Ma) [76] and the average genus diversification rate varied over time according to the *TreePar* analysis. Our results suggest that diversification was higher during the Oligocene and early Miocene compared to later geologic periods when net diversification rate decreased from 0.058 to 0.00013. During Oligo-Miocene, the oceanic circulation of the southern hemisphere was greatly modified by the northward movement of the Australian and South-American plates and the southward migration of the Antarctic plate [77]. These led to the formation of the circumpolar current, the formation of the Antarctic ice sheet [78]–[81] and global cooling, lowering sea levels and expanding emerged land masses and coastlines [82]. Furthermore, the collision of the Australian and Asian plates led to the emergence of new landmasses and tropical habitats suitable for colonization by shallow water species: the IAA [83]. The IAA formation modified equatorial currents, constraining seaways between the Pacific and Indian oceans. These changes in marine habitats induced massive species extinctions in many taxa and important changes in faunal compositions [84]. Based on our results, *Nerita* gastropods do not appear to have suffered from these global environmental changes since their diversity increased rapidly during this period (Figure 4). Increased availability of suitable habitats and the high spatial and temporal heterogeneity of the environment during Oligocene and early Miocene have likely modified the distribution and connectivity of populations and boosted diversification by increasing the opportunities for allopatric and peripatric speciation. The pattern we found is congruent with that of three other intertidal or shallow marine gastropod genera: *Conus, Echinolittorina* and *Turbo* [15]. The observed slowdown of diversification in these genera during late Miocene and Pliocene was interpreted as a consequence of limited speciation opportunities, due to the progressive filling of newly created niches [15]. Diversification rates in *Nerita* have likewise decreased slightly since the end of the Oligo-Miocene period. Contrastingly, the *N. undata* complex (originating from the IAA) presents a higher probability of diversification as shown by the RC test (p = 0.0076, α = 0.05). Originating during mid-Pliocene, this clade has diversified since this period in 11 robust genetic clades: 2 in the SWIO and 9 in the IAA. The detection of different geographically restricted lineages within species with Indo-Pacific distributions, like *N. albicilla* or *N. undata*, suggests that dispersal occurs at relatively small geographic scales, despite a high dispersal potential due to long-lived planktotrophic larvae. The spatial and temporal heterogeneity of the IAA region may enhance species diversification at small geographic scales by constantly modifying connectivity between populations for species with benthic adult and planktonic larval stages which are dependent on ocean currents and available habitats for settlement [85], [86]. Our results, like in other gastropod genera, support the centre-of-origin hypothesis for *Nerita*, the IAA presenting significantly more diversification events during the Oligocene.

### Isolation by Distance as a Driving Process of Diversification in the Indian Ocean

The genus *Nerita* being almost restricted to tropical rocky shores, its distribution is partly correlated to the existence of these particular habitats. Although the cryptic diversity of IAA *Nerita* can be explained by global climatic variations and environmental modifications over geological time, these factors do not explain the high proportion of endemic and cryptic lineages found within the SWIO. In this region, the volcanic activity (geological hotspot) started more than 65 Ma ago and created a North-South oriented chain of islands across the Indian Ocean: Laccadive islands (emergence: 65-60 Ma), Maldivian islands (60-50 Ma), Chagos archipelago (50-49 Ma), Mascarene plateau (48-31 Ma), Mauritius island (8 Ma) and finally Reunion Island (2 Ma) [87], [88]. During sea level low stands, these islands represented large landmasses, particularly during the Miocene. Various terrestrial clades used those multiple islands as stepping-stones to colonize the SWIO, while subsequent sea level rises facilitated secondary isolation and speciation [24]. Thus, colonization events of SWIO by Asian and IAA species favoured terrestrial speciation due to the action of geologic/climatic events throughout this period.

This model seems to apply to Indian Ocean *Nerita* species as well. Our hypothesis is supported by the old asynchronous divergence, ranging from 21 to 5 Ma (the Mascarene plateau emerged during this period) of three endemic species from their closest parent species living in the IAA region: *N. aterrima, N. magdalenae, N. umlaasiana*. Nowadays, there is little connectivity between the western and eastern Indian Ocean populations for a wide range of marine taxa [34], [89]. However, throughout the Miocene and assuming no major changes in ocean currents compared to nowadays, the intermittent emergence of volcanic landmasses and new rocky shores in SWIO may have permitted larval colonization from IAA *Nerita* populations. Without constant larval input due to sea level variations changing distances between populations of IAA and SWIO, newly settled populations diverged and formed new species by allopatry or peripatry. Ecological transition has not played a role in the formation of SWIO *Nerita* species as all sister species-pairs occupy the same ecological niches: lower littoral for *N. albicilla*-*N. sanguinolenta*; mid-littoral for *N. textilis*-*N. exuvia*; upper littoral for the pairs *N. magdalenae*-*N. costata* and *N. quadricolor*-*N. spengleriana*; supra-littoral for *N. insculpta*-*N. umlaasiana* [16]. Ecological conservatism have been identified in other intertidal gastropods, e.g., sister species of *Echinolittorina* remain allopatric for millions of years without changing their habitat preferences [33]. Ecological conservatism during diversification has also been documented in other taxa, such as coral reef fishes [90]. For a temperate terrestrial gastropod (*Arion subfuscus*), the habitat fidelity over time (as evidenced by the persistence of allopatry) has even contributed to the increase of lineage accumulation during the past glacial maximum [91].

Therefore SWIO *Nerita* endemics followed a “terrestrial” diversification pattern in the region and formed due to the synergy of several abiotic factors: the presence of an active geological hotspot and sea level variations, favouring colonization of *Nerita* populations from the IAA and subsequent genetic isolation. Changes in ocean circulation may have also played a role, but modelling currents at small geographical scale through geological time seems presently not feasible.

## Conclusions

This study joins the increasing number of publications linking geological history and diversification processes to explore biogeographic patterns. It brings attention to the role of geologic events and climatic variations modifying colonization opportunities. It further highlights the strong influence of allopatric and peripatric speciation processes in establishing intertidal gastropods diversity patterns, depending on the presence of islands to maintain their presence across oceanic basins and thus exhibiting a “terrestrial diversification pattern”. The link between the IAA and SWIO intertidal biodiversity is here evidence and it would be interesting to compare this pattern with other SWIO rocky shore taxa, in order to assess the prevalence of our results among a wider variety of organisms.

## Supporting Information

Table S1 **List of species used in this study, sampling zone, biogeographic region and Genbank accession numbers for CO1 and 16S genes.** CPO: Central Pacific Ocean; EAO: East Atlantic Ocean; EPO: East Pacific Ocean; IAA: Indo-Australian archipelago; NIO: North Indian Ocean; RS: Red Sea; SEIO: Southeast Indian Ocean; SWIO: Southwest Indian Ocean; SWPO: Southwest Pacific Ocean; WPO: West Pacific Ocean; WAO: West Atlantic Ocean;.
(XLSX)

Table S2 **Tests used on the final chronogram with assumptions and alternative hypotheses.**
(XLSX)

Table S3 **Diversification models tested with corresponding lineage-through-time plots.**
(XLSX)
