# Correction: Evolutionary Dynamics in the Southwest Indian Ocean Marine Biodiversity Hotspot: A Perspective from the Rocky Shore Gastropod Genus Nerita

**DOI:** 10.1371/journal.pone.0103393

**Published:** 2014-07-17

**Authors:** The PLOS ONE Staff

Figure 1 is incorrect. Please see the correct Figure 1 here.

**Figure 1:**
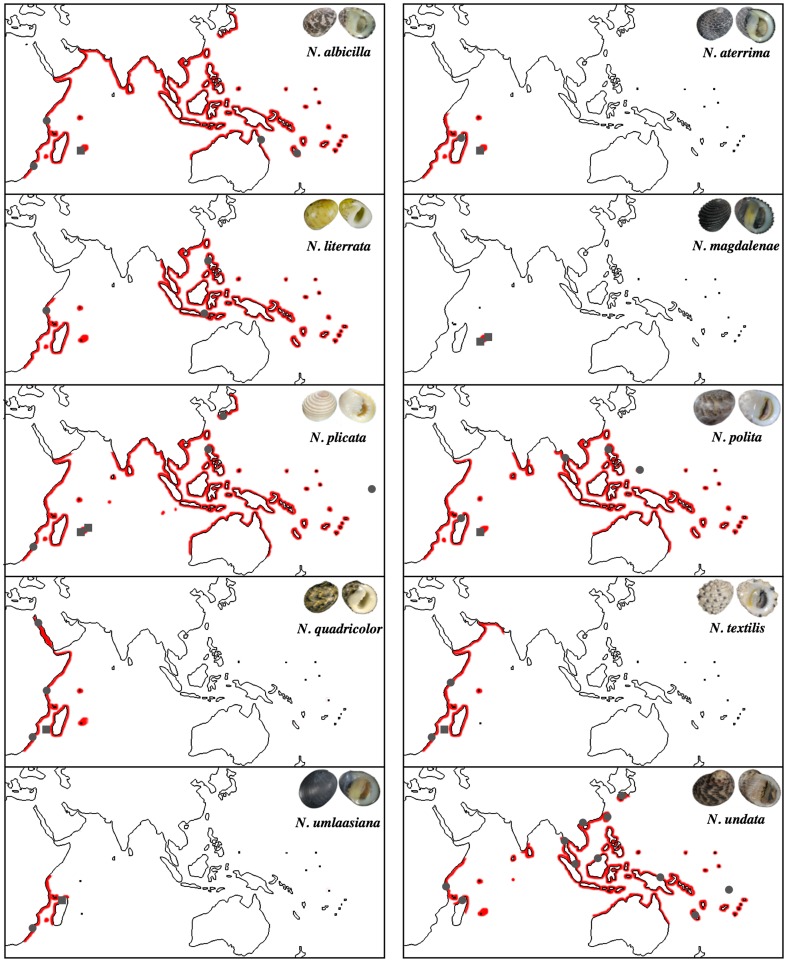
Geographic distribution of *Nerita* species living in the South-Western Indian Ocean, adapted from [16]. Grey dots indicate sampling sites from [16]; grey squares indicate sampling sites of new haplotypes.
